# Acute Kidney Injury and Pediatric Bone Health

**DOI:** 10.3389/fped.2020.635628

**Published:** 2021-02-09

**Authors:** Anisha Hegde, Michelle R. Denburg, Dorey A. Glenn

**Affiliations:** ^1^Department of Pediatrics, University of North Carolina Hospitals, Chapel Hill, NC, United States; ^2^Division of Nephrology, The Children's Hospital of Philadelphia, Perelman School of Medicine, University of Pennsylvania, Philadelphia, PA, United States; ^3^Division of Nephrology, University of North Carolina School of Medicine, Chapel Hill, NC, United States

**Keywords:** AKI, skeleton, parathyroid hormone, fibroblast growth factor 23, klotho, vitamin D, mineral, inflammation

## Abstract

Acute kidney injury (AKI) has been associated with deleterious impacts on a variety of body systems. While AKI is often accompanied by dysregulation of mineral metabolism—including alterations in calcium, phosphate, vitamin D, parathyroid hormone, fibroblast growth factor 23, and klotho—its direct effects on the skeletal system of children and adolescents remain largely unexplored. In this review, the pathophysiology of dysregulated mineral metabolism in AKI and its potential effects on skeletal health are discussed, including data associating AKI with fracture risk.

## Introduction

Acute kidney injury (AKI) results from a spectrum of insults that lead to a sudden decrease in kidney function and is responsible for significant morbidity and mortality (1–3). The worldwide incidence of pediatric AKI in hospital settings ranges from 26.9 to 41.3% (2, 4). Associated mortality ranges from 8.8 to 21% and is inversely related to gross national income per capita (2, 4, 5). AKI is accompanied by systemic inflammation (6) and has been associated with distant-organ injury—including injury to the lungs (7–9), heart (10), intestines (11), liver (11, 12), and brain (13)—that contributes to short- and long-term adverse patient outcomes (14, 15). AKI is frequently accompanied by dysregulated mineral metabolism (16), supporting the hypothesis that AKI may also be accompanied by alterations in skeletal structure and function. Commonly encountered dysregulation in mineral metabolism during AKI includes hypocalcemia, hyperphosphatemia, elevated parathyroid hormone (PTH), decreased 1,25-dihydroxyvitamin D (1,25D), elevated fibroblast growth factor 23 (FGF23), and decreased klotho. These patterns are similar to those seen in individuals with chronic kidney disease–mineral bone disorder (CKD-MBD), a condition known to alter skeletal and vascular biology. Compared to CKD-MBD, much less is known about the effects of mineral dysregulation and inflammation in AKI on short- and long-term skeletal health (16–18).

Identifying independent risk factors for skeletal injury in AKI is challenging for a number of reasons. First, AKI is a heterogeneous diagnosis with a myriad of inciting and exacerbating factors. Second, short-term biomarkers of skeletal injury are lacking, and the timeframe over which skeletal outcomes occur is long. Nonetheless, in this review, we aim to summarize the existing literature in three areas: (1) current data on fracture risk following AKI; (2) potential mechanisms of dysregulation in AKI, including inflammation, which could impact skeletal health; and (3) areas for further research.

## Fracture Risk and AKI

There is a striking paucity of literature exploring the association between AKI and risk of skeletal fracture. A single population-based matched cohort study conducted among 448 Taiwanese adult patients who survived dialysis-requiring AKI and 1,792 controls demonstrated a 1.25-fold increased risk of bone fracture (*p* = 0.049) in the AKI recovery group, even after controlling for progression to end-stage renal disease (19). Enrollees were identified through national database entries over 8 years and were prospectively followed for at least 1 year after hospital discharge. Incidence of bone fracture was 320 per 10,000 person-years in the AKI recovery group and 93 per 10,000 person-years in the control group (19). Patients with skeletal fracture also experienced increased long-term mortality (hazard ratio = 1.43, 95% confidence interval = 1.19–1.71, *p* < 0.001) (19). This is the only study to date that has evaluated long-term impacts of AKI on risk of skeletal fracture. Individuals with non–dialysis-dependent AKI and individuals younger than 18 years were not included in the study. These findings should be replicated in broader cohorts of adults with milder degrees of AKI and in children, as mineral dysregulation after AKI has been demonstrated in both groups (16, 20). There is also little published literature describing histologic changes in bone during or following AKI, with only one report describing findings from bone biopsy, including mild increases in bone resorption without increased osteoid (21).

## Mineral Dysregulation and AKI

### Hypocalcemia and Hyperphosphatemia

**Figure 1:**
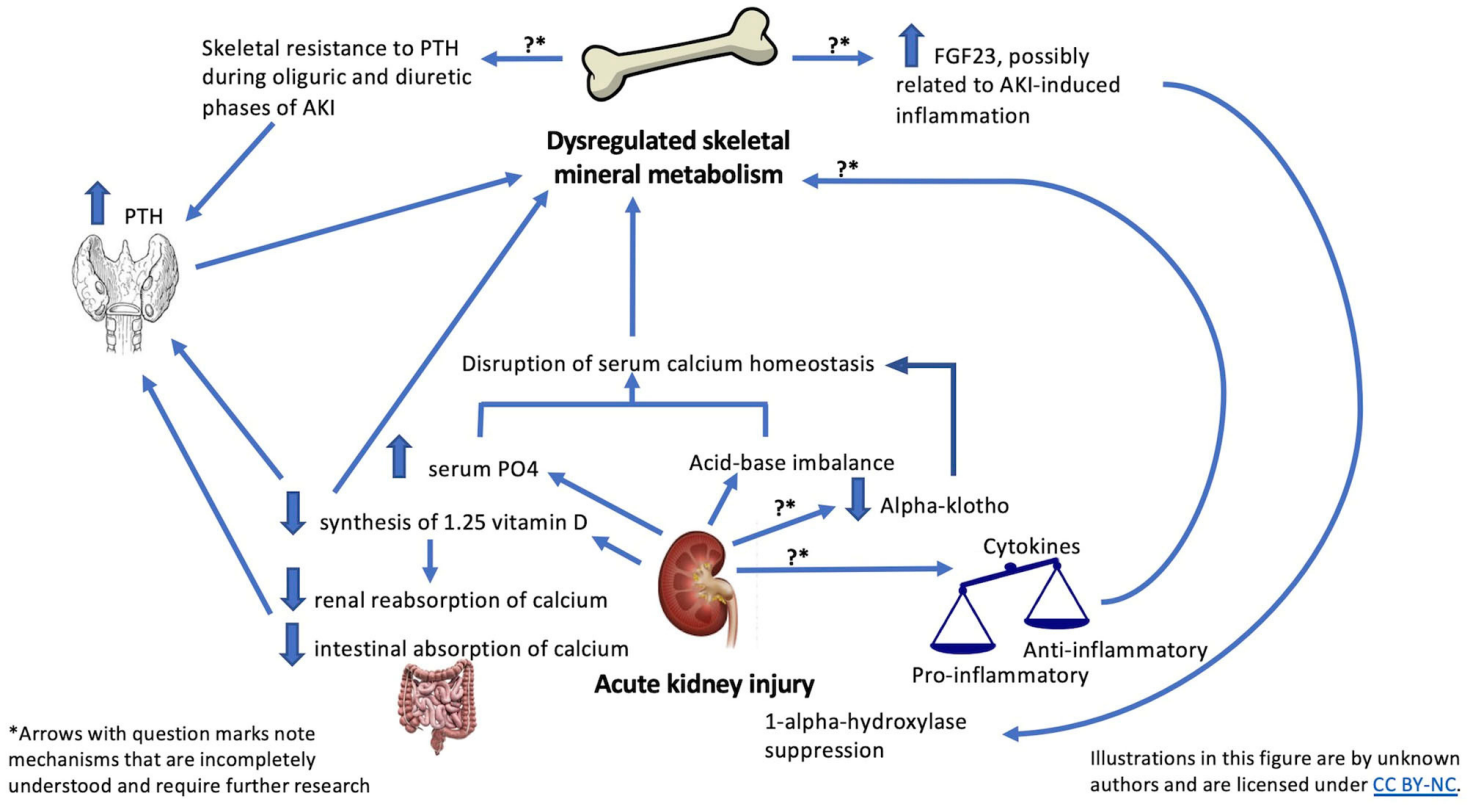
Summary of possible mechanisms linking AKI and skeletal outcomes.

The majority of total body calcium and phosphate is stored as skeletal hydroxyapatite, with extracellular calcium representing ~1% and extracellular phosphate representing <1% of total body stores (22, 23). Extracellular calcium and phosphate concentrations are primarily regulated by three hormones: 1,25D, PTH, and FGF23 [Figure 1; (24)]. Dysregulation of calcium and phosphate homeostasis is a consistent finding in studies of adult patients with varying severities of AKI (25–33). In a study of 400 critically ill adults with AKI, patients had a median phosphate level of 5.2 mg/dL with an interquartile range of 4.0–6.7 mg/dL (29). Additionally, multiple studies comparing mineral metabolism in patients with AKI to hospitalized control groups without AKI demonstrated significantly higher phosphate levels in AKI groups (28, 30, 31, 33). For example, in a cohort of 51 children who underwent elective cardiac surgery, serum phosphate increased significantly over a 24-h period in those who developed AKI compared to those who did not (*p* = 0.01) (34).

Of the aforementioned case-control studies that reported significantly elevated phosphate levels, two measured serum calcium levels. Leaf et al. reported significantly lower calcium levels in 30 adult patients with all-cause AKI compared to 30 patients without AKI (*p* = 0.004) (30). Patients with AKI had a serum calcium interquartile range of 7.5–8.6 mg/dL, and patients without AKI had an interquartile range of 8.1–9.0 mg/dL (30). In contrast, Zhang et al. did not detect a statistically significant difference in ionized calcium levels between critically ill adult patients with and without AKI (*p* = 0.41) (28). Patients with and without AKI had mean ionized calcium levels of 1.19 ± 0.1 mmol/L and 1.15 ± 0.08, respectively. Both studies are limited by small sample size, although differing findings could be explained by heterogeneous study populations (all hospitalized patients vs. only critically ill patients) and methods of measuring calcium (total serum vs. ionized). Hypocalcemia would be expected to follow significant hyperphosphatemia as elevated serum phosphate sequesters calcium. However, patients who are critically ill are also more likely to have aberrations in albumin and acid–base balance (22), causing a potential change in ionized calcium but not in total serum calcium. In addition to affecting ionized calcium levels, metabolic acidosis *in vitro* has been demonstrated to directly trigger osteoblast inhibition and osteoclast stimulation in mouse models (35).

Hypocalcemia and hyperphosphatemia may have acute as well as subacute clinical relevance (22). Acute hypocalcemia has well-described effects on decreasing peripheral vascular resistance (36), decreasing myocardial contractility (37), and increasing neuromuscular reactivity (22). Further research is needed to better understand the clinical impact that mineral alterations in AKI have on skeletal health, particularly among children undergoing rapid skeletal growth.

## Mechanisms of Mineral Dysregulation in AKI

### 1,25 Vitamin D

Alteration in regulatory hormones contributes to calcium and phosphate derangements following AKI. Decreased levels of 1,25D, and less frequently its precursor 25-hydroxyvitamin D (25D), have been described in multiple adult AKI cohorts, including in patients status post-cardiac surgery (26), those with rhabdomyolysis (27), and those with critical illness (29). There is growing evidence in adults that a history of critical illness is itself a strong risk factor for osteopenia and osteoporosis likely due to a combination of inflammation, undernutrition, immobilization, and vitamin D deficiency (38)—all of which are compounded by AKI. Similarly, vitamin D deficiency is common in critically ill children, with a pooled prevalence of 54.6% in a recent systematic review of hospitalized children with acute or critical conditions (39). Vitamin D deficiency, especially in critically ill patients, is linked to increased risk of AKI and mortality (40, 41). This link underscores the salience of investigating how vitamin D dysregulation during AKI superimposed on frequently reported baseline vitamin D deficiency may be contributing to adverse outcomes, including adverse skeletal outcomes.

Studies comparing 1,25D and 25D levels in adults with AKI to hospitalized controls without AKI have demonstrated significantly lower levels of 1,25D but not 25D (31, 42, 43). However, Tingting et al. reported lower levels of both 1,25D (*p* < 0.0001) and 25D (*p* < 0.0001) among 34 patients with critical illness and AKI compared to 12 healthy controls (44). Demographic differences in gender, age, and race between groups did not rise to statistical significance (44). This study's findings are perhaps explained by their use of healthy controls, as studies utilizing hospitalized controls have not shown differences in 25D, suggesting that low 25D may be a marker of global disease severity more so than of acute mineral dysregulation. Comparatively, Lai et al. enrolled 200 patients with AKI, 13 critically ill patients without AKI, and 17 healthy participants and reported that 1,25D levels but not 25D levels were significantly lower in the AKI group vs. the other groups (42). Taken together, these data suggest that AKI is associated with suppression of 1,25D and variable changes in 25D (16).

1,25D deficiency contributes to decreased intestinal absorption of calcium and decreased renal reabsorption of calcium (24, 45). These changes in AKI are thought to be mediated by decreased renal synthesis of 1,25D secondary to proximal tubule injury or from FGF23-induced suppression of 1-α-hydroxylase (46). Substrate delivery of 25D to the proximal tubule in AKI may also decrease because of lower levels of circulating vitamin D–binding protein (DBP), decreased filtration of 25D-DBP complexes due to reduced glomerular filtration rate (GFR), and decreased uptake of these complexes for processing due to proximal tubule injury (16).

Altered vitamin D metabolism in AKI, in addition to vitamin D deficiency potentially present on admission or attributed to critical illness, may be clinically relevant in multiple ways. Vitamin D plays a key role in skeletal health, mitigating fracture risk, and optimizing bone density and content through direct mineral regulation (47). 1,25D, through binding to its nuclear receptor, may also play a role in immune and inflammatory regulation (48). Even though vitamin D deficiency in AKI is likely transient, resolving with renal recovery (26, 27, 49), it has been associated with endothelial dysfunction (50), oxidative stress, and inflammation—both in the renal microvasculature and in distant organs (14, 51, 52). Arfian et al. demonstrated renoprotective effects of vitamin D—including reducing myofibroblasts and renal inflammation—in mice with kidney ischemia–reperfusion injuries (IRIs), although protective effects of vitamin D on distant organs such as bone following AKI have not yet been elucidated (52).

### Parathyroid Hormone

PTH is the primary regulator of serum calcium, and elevated PTH levels have been frequently reported in patients with AKI (16, 17, 26, 27, 29). Studies have consistently demonstrated significantly elevated PTH in individuals with AKI compared to healthy controls (44, 53, 54). These elevations are thought to be secondary to hypocalcemia and low serum 1,25D levels as discussed above (24). Upregulation of the parathyroid calcium-sensing receptor due to acute inflammation may additionally affect the calcium-PTH set-point (16, 24).

Skeletal and renal resistance to PTH has been described in humans following AKI (25, 29, 30), potentially contributing to dysregulated hormonal control of calcium and phosphate. In patients with CKD, skeletal resistance to PTH is thought to be secondary to downregulated PTH receptors (16); however, specific mechanisms of PTH resistance in AKI have not been studied. Massry et al. demonstrated that patients with AKI failed to respond appropriately to exogenous PTH infusion during their oliguric and diuretic phases of AKI but produced appropriate increases in serum calcium once renal function recovered (25).

Thus, skeletal resistance to PTH in AKI is likely a transient phenomenon, as is elevated PTH (26, 30). For example, in a study comparing PTH levels in hospitalized adults with and without all-cause AKI, PTH levels were initially higher in the AKI group (*p* = 0.004) compared to the non-AKI group but were no longer different by day 5 of enrollment (*p* = 0.56) (30). Additional studies of hospitalized adults with and without AKI following cardiac surgery and critical illness (28, 31) have reported that PTH was comparatively elevated (and occasionally severely, with PTH >250 mg/dL) in AKI groups but did not rise to statistical significance. Small sample sizes and heterogeneous patient populations may explain these inconsistent findings. Likewise, findings may be confounded by elevations of PTH due to critical illness (55, 56).

### Fibroblast Growth Factor 23 and Klotho

FGF23 is a bone-derived protein, produced by osteoblasts and osteocytes and known to regulate vitamin D metabolism and phosphate homeostasis (57–59). FGF23 was first identified in patients with tumor-induced osteomalacia and mineralization defects and has since become an important link connecting renal and skeletal physiology (60). Studies have consistently demonstrated that FGF23 levels increase rapidly after onset of AKI (28–31, 61–66). In mouse models of folic acid–induced AKI, FGF23 levels have been shown to rise within 1 h following AKI (62). In adults with AKI after cardiac surgery, FGF23 levels demonstrated more than a 15-fold increase at 24 h post-surgery (62).

While there is strong evidence of a role for FGF23 in mediating phosphate and vitamin D dysregulation in early-stage CKD (67), the effects of FGF23 on skeletal health following AKI have not been studied (61, 68). Rise in FGF23 following AKI appears to be independent of PTH and 1,25D levels (20, 58, 62, 69), but resultant effects of FGF23 on PTH and 1,25D levels in AKI have yet to be conclusively demonstrated. FGF23 is also thought to play a role in the inflammatory response following AKI (58). In mice with folic acid–induced AKI, very high *FGF23* mRNA expression was detected in thymus and spleen, suggesting that FGF23 may be associated with increased *TNF* expression and elevated inflammatory cytokine levels through effects on lymphoid organs (58).

FGF23 elevation is likely altered in a timeframe consistent with reduced renal function. For example, in 32 pediatric patients who underwent cardiac surgery requiring cardiopulmonary bypass, those children who developed post-operative AKI demonstrated a significant increase in intact FGF23 at 2 h post-reperfusion compared to those who did not develop AKI (*p* = 0.04). At 48 h, intact FGF23 levels between these groups were not significantly different (*p* = 0.19), although C-terminal FGF23 levels were significantly elevated (*p* = 0.006) in the AKI group (20). Animal models of AKI due to folic acid nephropathy have demonstrated increased FGF23 expression in femur lysates, but the factors responsible for this elevation in bone during a period of acutely reduced renal function have not been identified (58, 62, 64).

α-Klotho, a transmembrane protein mainly produced by renal tubular epithelial cells, plays a critical role in FGF23 receptor binding (16) and, through its actions on the kidney and parathyroid glands, plays an important role in mineral metabolism (64, 70, 71). Its extracellular domain can modulate renal calcium and potassium absorption independently of FGF23 (16, 72) in the distal tubule and can be cleaved into the circulation, facilitating distant organ effects of FGF23 (64). In CKD mouse models, klotho has been shown to decrease ectopic calcification (including soft tissue calcification), likely through tight coregulation of phosphorous with FGF23 (70). Klotho deficiency has been demonstrated in animal models of AKI (73, 74), although studies in humans are lacking (64, 71, 73, 75–78). Cytokines produced during AKI, including tumor necrosis factor α (TNF-α), may downregulate renal expression of klotho (79). No studies have yet examined whether klotho deficiency may contribute to aberrant calcification in AKI.

### Inflammation in AKI

Acute intrarenal and systemic inflammation, including the release of proinflammatory cytokines, has been well-described following AKI (6, 7, 11–14, 80–84). Inflammation is a crucial biologic response for eliminating pathogens and repairing injured tissue. However, the balance between proinflammatory and anti-inflammatory responses is often abnormal in AKI (83). In renal IRI, cellular damage triggers an inflammatory response that includes oxidative stress in renal tubular epithelial cells, necrotic cells that release a variety of molecules to signal damage, and cytokine release from activated renal parenchymal cells and dendritic cells that recruit innate and adaptive immune mediators (83).

Increased serum levels of proinflammatory cytokines, including TNF-α, interleukin 6 (IL-6), and IL-8, have been described during AKI in animal models of renal IRI (80). These animal models have demonstrated dramatic increases in plasma cytokine levels compared to non-IRI animal models, including sham surgery and bilateral nephrectomy (80). Similar increases in cytokine levels have been noted in trauma patients with AKI compared to those without AKI early on in their hospital courses (80). Circulating cytokines during AKI have been shown to impact distant organs, including the brain (13), liver (11), and lungs (8). In addition to increased cytokine production, experimental models indicate that cytokine clearance is also decreased in AKI (80).

Although direct effects of proinflammatory cytokines specifically on bone during AKI have not been studied, proinflammatory cytokines have been associated with adverse effects on bone formation and resorption [Figure 1; (85–87)] For example, TNF-α and IL-6 have been shown to activate the parathyroid calcium-sensing receptor (88) and to inhibit renal expression of 1-α-hydroxylase (89), which could contribute to hormonal dysregulation of mineral metabolism in AKI. TNF-α has been reported to inhibit *PHEX* gene expression. *PHEX* gene is expressed mostly in osteoblasts, and loss of PHEX function has been linked to defective mineralization (90). Additionally, data from the Women's Health Initiative Study (91) suggest that TNF-α plays a role in mediating fracture risk, as association between estimated GFR and fracture risk was eliminated after adjustment for TNF-α receptor levels.

Anti-inflammatory cytokines are also increased in AKI (83). IL-10 is an anti-inflammatory cytokine that may have osteoprotective effects and has emerged along with IL-6 as a key player in signaling distant effects of acute renal inflammation (81). IL-10 is produced by T-regulatory cells (60) that *in vitro* directly inhibit osteoclast activity, including their differentiation and function, and *in vivo* have been shown to protect against TNF-α-induced bone loss in mice (92). The net outcome of this interplay between osteotoxic and osteoprotective factors on bone in the setting of AKI has yet to be determined (83).

Transcriptomics research in a murine model of ischemic AKI identified increased levels of IL-10 and IL-6 in lung tissue after AKI, in addition to global transcriptomic changes and histologic injury (81). Similar to the lung, the skeletal system has an extensive capillary network, including in the metabolically active skeletal system of children and adolescents undergoing periods of rapid growth (93). Thus, the relationship of inflammation in AKI and its potential direct consequences on bone health is an area ripe for additional investigation (80, 84).

## Areas for Future Research

1. Epidemiological studies are needed to further characterize the burden of and risk factors for skeletal complications, including fractures, in patients with AKI. These studies should include pediatric patients, who are experiencing AKI during times of bone mineral accrual and linear growth (24).
2. Larger studies are needed to better characterize which aspects of dysregulated mineral metabolism, if any, persist after renal recovery following AKI.
3. The use of bone imaging (dual energy x-ray absorptiometry / high resolution peripheral quantitative computed tomography) lends itself to investigations into whether changes in bone structure and microarchitecture are seen in the acute or subacute phases of AKI.
4. Further investigation is needed regarding the extent to which systemic vascular and inflammatory changes detected following AKI, including vitamin D–associated changes, alter bone epigenetics and transcriptomics and might contribute to effects on long-term skeletal health. Metabolomics studies in AKI may identify downstream targets relevant for additional investigation (10, 94).
5. Although the pathophysiology of AKI generally involves a common cascade of inflammation secondary to ischemia, reperfusion, cell injury, and cell death, AKI remains a clinically heterogeneous diagnosis with varying therapies. The potential compounding effect of frequently used therapies in AKI, such as diuretics and other medications (87), on mineral dysregulation and bone health has not been evaluated.

## Author Contributions

AH and DG contributed to the literature review and layout of this article. AH wrote the first draft of the manuscript, which was revised by DG. AH, DG, and MD contributed to final manuscript revision and have approved the submitted version. All authors contributed to the article and approved the submitted version.

## Conflict of Interest

MD reports funding from Mallinckrodt Pharmaceuticals.

The remaining authors declare that the research was conducted in the absence of any commercial or financial relationships that could be construed as a potential conflict of interest.

## References

[1] Goldstein SL Adrogue HE Chapter 365: Acute kidney injury. In: McInerny T Adam H Campbell D DeWitt T Foy J Kamat D, editors. American Academy of Pediatrics Textbook of Pediatric Care. 2nd ed (2017). Available online at: https://pediatriccare.solutions.aap.org/chapter.aspx?sectionid=138300635&bookid=1626 (accessed December 01, 2020).

[2] Lameire N Van Biesen W Vanholder R. Epidemiology of acute kidney injury in children worldwide, including developing countries. Pediatr Nephrol. (2017) 32:1301–14. 10.1007/s00467-016-3433-2 27307245

[3] Khwaja A. KDIGO clinical practice guidelines for acute kidney injury. Nephron Clin Pract. (2012) 120:c179–84. 10.1159/000339789 22890468

[4] Susantitaphong P Cruz DN Cerda J Abulfaraj M Alqahtani F Koulouridis I. World incidence of AKI: A meta-analysis. Clin J Am Soc Nephrol. (2013) 8:1482–93. 10.2215/CJN.00710113 23744003 PMC3805065

[5] Fortrie G De Geus HRH Betjes MGH. The aftermath of acute kidney injury: a narrative review of long-term mortality and renal function. Crit Care. (2019) 23:24. 10.1186/s13054-019-2314-z 30678696 PMC6346585

[6] Gyurászová M Kovalčíková AG Renczés E Kmetová K Celec P Bábíčková J. Oxidative stress in animal models of acute and chronic renal failure. Dis Markers. (2019) 2019:8690805. 10.1155/2019/8690805 30886657 PMC6388331

[7] Andres-Hernando A Altmann C Bhargava R Okamura K Bacalja J Hunter B. Prolonged acute kidney injury exacerbates lung inflammation at 7 days post-acute kidney injury. Physiol Rep. (2014) 2:e12084. 10.14814/phy2.12084 25052489 PMC4187574

[8] Faubel S Edelstein CL. Mechanisms and mediators of lung injury after acute kidney injury. Nat Rev Nephrol. (2016) 12:48–60. 10.1038/nrneph.2015.158 26434402

[9] Teixeira JP Ambruso S Griffin BR Faubel S. Pulmonary consequences of acute kidney injury. Semin Nephrol. (2019) 39:3–16. 10.1016/j.semnephrol.2018.10.001 30606405

[10] Fox BM Gil HW Kirkbride-Romeo L Bagchi RA Wennersten SA Haefner KR. Metabolomics assessment reveals oxidative stress and altered energy production in the heart after ischemic acute kidney injury in mice. Kidney Int. (2019) 95:590–610. 10.1016/j.kint.2018.10.020 30709662 PMC6564679

[11] Park SW Chen SW Kim M Brown KM Kolls JK D'Agati VD. Cytokines induce small intestine and liver injury after renal ischemia or nephrectomy. Lab Investig. (2011) 91:63–84. 10.1038/labinvest.2010.151 20697374 PMC2991383

[12] Shang Y Madduma Hewage S Wijerathne CUB Siow YL Isaak CK Karmin O. Kidney ischemia-reperfusion elicits acute liver injury and inflammatory response. Front Med. (2020) 7:201. 10.3389/fmed.2020.00201 32582723 PMC7280447

[13] Liu M Liang Y Chigurupati S Lathia JD Pletnikov M Sun Z. Acute kidney injury leads to inflammation and functional changes in the brain. J Am Soc Nephrol. (2008) 19:1360–70. 10.1681/ASN.2007080901 18385426 PMC2440297

[14] Druml W. Systemic consequences of acute kidney injury. Curr Opin Crit Care. (2014) 20:613–9. 10.1097/MCC.0000000000000150 25259720

[15] Uber AM Sutherland SM. Acute kidney injury in hospitalized children: consequences and outcomes. Pediatr Nephrol. (2020) 35:213–20. 10.1007/s00467-018-4128-7 30386936 PMC7223774

[16] Leaf DE Christov M Dysregulated mineral metabolism in AKI. Semin Nephrol. (2019) 39:41–56. 10.1016/j.semnephrol.2018.10.004 30606407

[17] Wesseling-Perry K Bone disease in pediatric chronic kidney disease. Pediatr Nephrol. (2013) 28:569–76. 10.1007/s00467-012-2324-4 23064662 PMC3594120

[18] Yang T Wang W Tang X Shi P Zhang L Yu W. Association between mineral and bone disorder in patients with acute kidney injury following cardiac surgery and adverse outcomes. BMC Nephrol. (2019) 20:369. 10.1186/s12882-019-1572-y 31615432 PMC6794865

[19] Wang WJ Chao C Ter Huang YC Wang CY Chang CH Huang TM. The impact of acute kidney injury with temporary dialysis on the risk of fracture. J Bone Miner Res. (2014) 29:676–84. 10.1002/jbmr.2061 23929760

[20] Hanudel MR Wesseling-Perry K Gales B Ramos G Campbell V Ethridge K. Effects of acute kidney injury and chronic hypoxemia on fibroblast growth factor 23 levels in pediatric cardiac surgery patients. Pediatr Nephrol. (2016) 31:661–9. 10.1007/s00467-015-3257-5 26525200 PMC4766020

[21] Zech P Bone biopsies in patients with acute renal failure. In: Proceedings of the International Symposium “The Kidney and Calcium” (Les Trois Epis) (1972).

[22] Moe SM. Disorders involving calcium, phosphorus, and magnesium. Prim Care. (2008) 35:215. 10.1016/j.pop.2008.01.007 18486714 PMC2486454

[23] Fukumoto S Phosphate metabolism and vitamin D. Bonekey Rep. (2014) 3:497 10.1038/bonekey.2013.231 24605214 PMC3944128

[24] Underland L Markowitz M Gensure R Calcium and phosphate hormones: vitamin D, parathyroid hormone, and fibroblast growth factor 23 education gap. Pediatrics Rev. (2020) 41:3–11. 10.1542/pir.2018-0065 31894068

[25] Massry SG Arieff AI Coburn JW Palmieri G Kleeman CR. Divalent ion metabolism in patients with acute renal failure: studies on the mechanism of hypocalcemia. Kidney Int. (1974) 5:437–445. 10.1038/ki.1974.62 4421787

[26] Pietrek J Kokot F Kuska J. Serum 25-hydroxyvitamin D and parathyroid hormone in patients with acute renal failure. Kidney Int. (1978) 13:178–85. 10.1038/ki.1978.25 713278

[27] Llach F Felsenfeld AJ HM. The pathophysiology of altered calcium metabolism in rhabdomyolysis-induced acute renal failure. N Engl J Med. (1981) 305:117–23. 6894630 10.1056/NEJM198107163050301

[28] Zhang M Hsu R Hsu C Kordesch K Nicasio E Cortez A. FGF-23 and PTH levels in patients with acute kidney injury: a cross-sectional case series study. Ann Intensive Care. (2011) 1:21. 10.1186/2110-5820-1-21 21906363 PMC3224491

[29] Leaf DE Siew ED Eisenga MF Singh K Mc Causland FR Srivastava A. Fibroblast growth factor 23 associates with death in critically ill patients. Clin J Am Soc Nephrol. (2018) 13:531–541. 10.2215/CJN.10810917 29519954 PMC5969465

[30] Leaf DE Wolf M Waikar SS Chase H Christov M Cremers S. FGF-23 levels in patients with AKI and risk of adverse outcomes. Clin J Am Soc Nephrol. (2012) 7:1217–23. 10.2215/CJN.00550112 22700885 PMC3408118

[31] Leaf DE Christov M Jüppner H Siew E Ikizler TA Bian A. Fibroblast growth factor 23 levels are elevated and associated with severe acute kidney injury and death following cardiac surgery. Kidney Int. (2016) 89:939–48. 10.1016/j.kint.2015.12.035 26924052 PMC4801748

[32] Jung SY Kim H Park S Jhee JH Yun HR Kim H. Electrolyte and mineral disturbances in septic acute kidney injury patients undergoing continuous renal replacement therapy. Medicine. (2016) 95:e4542. 10.1097/MD.0000000000004542 27603344 PMC5023866

[33] Saour M Zeroual N Ridolfo J Nogue E Picot M-C Gaudard P. Serum phosphate kinetics in acute kidney injury after cardiac surgery: an observational study. J Cardiothorac Vasc Anesth. (2020) 34: 2964–72. 10.1053/j.jvca.2020.05.023 32660927

[34] Burra V Nagaraja PS Singh NG Prabhakar V Manjunatha N. Early prediction of acute kidney injury using serum phosphorus as a biomarker in pediatric cardiac surgical patients. Ann Card Anaesth. (2018) 21:455–9. 10.4103/aca.ACA_14_18 30333349 PMC6206811

[35] Krieger NS Sessler NE Bushinsky DA. Acidosis inhibits osteoblastic and stimulates osteoclastic activity *in vitro*. Am J Physiol Physiol. (1992) 262(3 Pt 2):F442–8. 10.1152/ajprenal.1992.262.3.F442 1558161

[36] Scheidegger D Drop LJ Schellenberg J-C. Role of the systemic vasculature in the hemodynamic response to changes in plasma ionized calcium. Arch Surg. (1980). 115:206–11. 10.1001/archsurg.1980.01380020072017 6101538

[37] Szent-Györgyi AG Calcium regulation of muscle contraction. Biophys J. (1975) 15:707–23. 10.1016/S0006-3495(75)85849-8 806311 PMC1334730

[38] Rousseau AF Kerschan-Schindl K Scherkl M Amrein K. Bone metabolism and fracture risk during and after critical illness. Curr Opin Crit Care. (2020) 26:379–85. 10.1097/MCC.0000000000000734 32520810

[39] Cariolou M Cupp MA Evangelou E Tzoulaki I Berlanga-Taylor AJ. Importance of Vitamin D in acute and critically ill children with subgroup analyses of sepsis and respiratory tract infections: a systematic review and meta-analysis. BMJ Open. (2019) 9:e027666. 10.1136/bmjopen-2018-027666 31122993 PMC6538078

[40] Braun A Christopher K Vitamin D in acute kidney injury. Inflamm Allergy-Drug Targets. (2013) 12:262–72. 10.2174/18715281113129990044 23782211

[41] Braun AB Litonjua AA Moromizato T Gibbons FK Giovannucci E Christopher KB. Association of low serum 25-hydroxyvitamin D levels and acute kidney injury in the critically ill. Crit Care Med. (2012) 40:3170–9. 10.1097/CCM.0b013e318260c928 22975885

[42] Lai L Qian J Yang Y Xie Q You H Zhou Y. Is the serum vitamin D level at the time of hospital-acquired acute kidney injury diagnosis associated with prognosis? PLoS ONE. (2013) 8:e64964. 10.1371/journal.pone.0064964 23717679 PMC3661528

[43] Leaf DE Waikar SS Wolf M Cremers S Bhan I Stern L. Dysregulated mineral metabolism in patients with acute kidney injury and risk of adverse outcomes. Clin Endocrinol. (2013) 79:491–8. 10.1111/cen.12172 23414198 PMC3686895

[44] Tingting L Vijayan A Dusso A Jain S Coyne DW. Relationship of 1,25 dihydroxy vitamin D levels to clinical outcomes in critically ill patients with acute kidney injury. J Nephrol Ther. (2014) 5:190. 10.4172/2161-0959.1000190 26295008 PMC4540223

[45] Slatopolsky E Weerts C Thielan J Horst R Harter H Martin KJ Marked suppression of secondary hyperparathyroidism by intravenous administration of 1,25-dihydroxycholecalciferol in uremic patients. J Clin Invest. (1984) 74:2136–43. 10.1172/JCI111639 6549016 PMC425405

[46] Hsu CH Patel S Young EW Simpson RU. Production and metabolic clearance of calcitriol in acute renal failure. Kidney Int. (1988) 33:530–5. 10.1038/ki.1988.30 3361754

[47] Ross AC Manson JE Abrams SA Aloia JF Brannon PM Clinton SK. The 2011 report on dietary reference intakes for calcium and vitamin D from the Institute of Medicine: what clinicians need to know. J Clin Endocrinol Metab. (2011) 96:53–8. 10.1210/jc.2010-2704 21118827 PMC3046611

[48] Ramagopalan SV Heger A Berlanga AJ Maugeri NJ Lincoln MR Burrell A. A ChIP-seq defined genome-wide map of vitamin D receptor binding: associations with disease and evolution. Genome Res. (2010) 20:1352–60. 10.1101/gr.107920.110 20736230 PMC2945184

[49] Cameron LK Lei K Smith S Doyle NL Doyle JF Flynn K. Vitamin D levels in critically ill patients with acute kidney injury: a protocol for a prospective cohort study (VID-AKI). BMJ Open. (2017) 7:e016486. 10.1136/bmjopen-2017-016486 28706103 PMC5726075

[50] de Bragança AC Volpini RA Mehrotra P Andrade L Basile DP. Vitamin D deficiency contributes to vascular damage in sustained ischemic acute kidney injury. Physiol Rep. (2016) 4:e12829. 10.14814/phy2.12829 27369932 PMC4945834

[51] Reis NG Francescato HDC de Almeida LF da Silva CGA Costa RS Coimbra TM. Protective effect of calcitriol on rhabdomyolysis-induced acute kidney injury in rats. Sci Rep. (2019) 9:7090. 10.1038/s41598-019-43564-1 31068635 PMC6506495

[52] Arfian N Budiharjo S Prasetyo Wibisono D Ananda Wahyu Setyaningsih W Mansyur Romi M Laila Afifa An-nur Willya Saputri R. Vitamin D ameliorates kidney ischemia reperfusion injury via reduction of inflammation and myofibroblast expansion. Kobe J Med Sci. (2019) 65:E138–43. 10.24546/81012029 32201429 PMC7447095

[53] Druml W Schwarzenhofer M Apsner RHW Fat-soluble vitamins in patients with acute renal failure. Min Electrolyte Metab. (1998) 24:220–6. 10.1159/000057374 9554560

[54] Shieh SD Lin YF Lin SH LK. A prospective study of calcium metabolism in exertional heat stroke with rhabdomyolysis and acute renal failure. Nephron. (1995) 71:428–32. 8587623 10.1159/000188763

[55] Terzioglu-Usak S Elibol B Dalli T Guler C Aysan E Effect of restraint stress on plasma PTH concentration and its molecular targets expressions in Wistar rats. Int J Endocrinol Metab. (2018) 16:e66979 10.5812/ijem.66979 30464774 PMC6216602

[56] Zaloga GP Teres D Critical illness is associated with elevated parathyroid hormone. Crit Care. (2001) 5:P208 10.1186/cc1275

[57] Hu MC Shiizaki K Kuro-o M Moe OW. Fibroblast growth factor 23 and klotho: physiology and pathophysiology of an endocrine network of mineral metabolism. Annu Rev Physiol. (2013) 75:503–33. 10.1146/annurev-physiol-030212-183727 23398153 PMC3770142

[58] Egli-Spichtig D Zhang MYH Perwad F. Fibroblast growth factor 23 expression is increased in multiple organs in mice with folic acid-induced acute kidney injury. Front Physiol. (2018) 9:1494. 10.3389/fphys.2018.01494 30405444 PMC6206018

[59] Neyra JA Moe OW Hu MC Fibroblast growth factor 23 and acute kidney injury. Pediatr Nephrol. (2015) 30:1909–18. 10.1007/s00467-014-3006-1 25480729 PMC4458234

[60] Zaidi M Yuen T Sun L Rosen CJ Regulation of skeletal homeostasis. Endocr Rev. (2018) 39:701–18. 10.1210/er.2018-00050 29897433 PMC6173473

[61] Christov M Fibroblast growth factor 23 in acute kidney injury. Curr Opin Nephrol Hypertens. (2014) 23:340–5. 10.1097/01.mnh.0000447021.51722.2f 24848938 PMC4183160

[62] Christov M Waikar SS Pereira RC Havasi A Leaf DE Goltzman D. Plasma FGF23 levels increase rapidly after acute kidney injury. Kidney Int. (2013) 84:776–85. 10.1038/ki.2013.150 23657144 PMC3766419

[63] Leaf DE Jacob KA Srivastava A Chen ME Christov M Jüppner H. Fibroblast growth factor 23 levels associate with AKI and death in critical illness. J Am Soc Nephrol. (2017) 28:1877–85. 10.1681/ASN.2016080836 28028134 PMC5461795

[64] Christov M Neyra JA Gupta S Leaf DE. Fibroblast growth factor 23 and klotho in AKI. Semin Nephrol. (2019) 39:57–75. 10.1016/j.semnephrol.2018.10.005 30606408

[65] Fayed A Radwan WA Amin M Gamal A. Prediction of mortality and need for renal replacement therapy in patients of acute kidney injury using fibroblast growth factor 23. Saudi J Kidney Dis Transpl. (2019) 30:1044–51. 10.4103/1319-2442.270259 31696842

[66] Meurer M Höcherl K. Renal ischemia-reperfusion injury impairs renal calcium, magnesium, and phosphate handling in mice. Pflugers Arch Eur J Physiol. (2019) 471:901–14. 10.1007/s00424-019-02255-6 30685787

[67] Hasegawa H Nagano N Urakawa I Yamazaki Y Iijima K Fujita T. Direct evidence for a causative role of FGF23 in the abnormal renal phosphate handling and vitamin D metabolism in rats with early-stage chronic kidney disease. Kidney Int. (2010) 78:975–80. 10.1038/ki.2010.313 20844473

[68] Kronenberg H. Npt2a–the key to phosphate homeostasis. N Engl J Med. (2002) 347:1022–24. 10.1056/NEJMe020098 12324560

[69] Shimada T Hasegawa H Yamazaki Y Muto T Hino R Takeuchi Y. FGF-23 is a potent regulator of vitamin D metabolism and phosphate homeostasis. J Bone Miner Res. (2004) 19:429–35. 10.1359/JBMR.0301264 15040831

[70] Hu M-C Kuro-O M Moe OW Klotho and kidney disease. J Nephrol. (2010) 23:(Suppl. 16):S136–44. 10.1159/000346778 21170871 PMC3227531

[71] Castellano G Intini A Stasi A Divella C Gigante M Pontrelli P. Complement modulation of anti-aging factor klotho in ischemia/reperfusion injury and delayed graft function. Am J Transplant. (2016) 16:325–33. 10.1111/ajt.13415 26280899

[72] Imura A Tsuji Y Murata M Maeda R Kubota K Iwano A Alpha-klotho as a regulator of calcium homeostasis. Science. (2007) 316: 1615–8. 10.1126/science.1135901 17569864

[73] Hu MC Shi M Zhang J Quiones H Kuro-O M Moe OW Klotho deficiency is an early biomarker of renal ischemia-reperfusion injury and its replacement is protective. Kidney Int. (2010) 78:1240–51. 10.1038/ki.2010.328 20861825 PMC3237296

[74] Sugiura H Yoshida T Tsuchiya K Mitobe M Nishimura S Shirota S Klotho reduces apoptosis in experimental ischaemic acute renal failure. Nephrol Dial Transplant. (2005) 20:2636–45. 10.1093/ndt/gfi165 16204278

[75] Kim AJ Ro H Kim H Chang JH Lee HH Chung W. Klotho and S100A8/A9 as discriminative markers between pre-renal and intrinsic acute kidney injury. PLoS ONE. (2016) 11:e0147255. 10.1371/journal.pone.0147255 26799323 PMC4723127

[76] Seo MY Yang J Lee JY Kim K Kim SC Chang H. Renal klotho expression in patients with acute kidney injury is associated with the severity of the injury. Korean J Intern Med. (2015) 30:489–95. 10.3904/kjim.2015.30.4.489 26161015 PMC4497336

[77] Neyra JA Li X Mescia F Ortiz-Soriano V Adams-Huet B Pastor J. Urine klotho is lower in critically ill patients with versus without acute kidney injury and associates with major adverse kidney events. Crit Care Explor. (2019) 1:e0016. 10.1097/cce.0000000000000016 32123869 PMC7051168

[78] Seibert E Radler D Ulrich C Hanika S Fiedler R Girndt M. Serum klotho levels in acute kidney injury. Clin Nephrol. (2017) 87:173–9. 10.5414/CN108970 28157067

[79] Ruiz-Andres O Dolores Sanchez-Niño M Antonio Moreno J Ruiz-Ortega M Mario Ramos A Belen Sanz A. Downregulation of kidney protective factors by inflammation: role of transcription factors and epigenetic mechanisms. Am J Physiol Ren Physiol. (2016) 311:1329–40. 10.1152/ajprenal.00487.2016 27760772

[80] Singbartl K Formeck CL Kellum JA Kidney-immune system crosstalk in AKI. Semin Nephrol. (2019) 39:96–106. 10.1016/j.semnephrol.2018.10.007 30606411

[81] Grigoryev DN Liu M Hassoun HT Cheadle C Barnes KC Rabb H. The local and systemic inflammatory transcriptome after acute kidney injury. J Am Soc Nephrol. (2008) 19:547–58. 10.1681/ASN.2007040469 18235097 PMC2391061

[82] Ratliff BB Rabadi MM Vasko R Yasuda K Goligorsky MS. Messengers without borders: mediators of systemic inflammatory response in AKI. J Am Soc Nephrol. (2013) 24:529–36. 10.1681/ASN.2012060633 23349311

[83] Rabb H Griffin MD McKay DiB Swaminathan S Pickkers P Rosner MH. Inflammation in AKI: current understanding, key questions, and knowledge gaps. J Am Soc Nephrol. (2016) 27:371–9. 10.1681/ASN.2015030261 26561643 PMC4731128

[84] Darisipudi MN Knauf F. An update on the role of the inflammasomes in the pathogenesis of kidney diseases. Pediatr Nephrol. (2016) 31:535–44. 10.1007/s00467-015-3153-z 26178650

[85] Gilbert L He X Farmer P Rubin J Drissi H Van Wijnen AJ. Expression of the osteoblast differentiation factor RUNX2 (Cbfa1/AML3/Pebp2αA) is inhibited by tumor necrosis factor-α. J Biol Chem. (2002) 277:2695–701. 10.1074/jbc.M106339200 11723115

[86] Ahuja SS Zhao S Bellido T Plotkin LI Jimenez F Bonewald LF. CD40 ligand blocks apoptosis induced by tumor necrosis factor α, glucocorticoids, and etoposide in osteoblasts and the osteocyte-like cell line murine long bone osteocyte-Y4. Endocrinology. (2003) 144:1761–9. 10.1210/en.2002-221136 12697681

[87] Glenn DA Denburg MR. Bone health in glomerular kidney disease. Curr Osteoporos Rep. (2019) 17:570–9. 10.1007/s11914-019-00531-z 31734906 PMC7153693

[88] Hendy GN Canaff L. Calcium-sensing receptor, proinflammatory cytokines and calcium homeostasis. Semin Cell Dev Biol. (2016) 49:37–43. 10.1016/j.semcdb.2015.11.006 26612442

[89] Liu N Nguyen L Chun RF Lagishetty V Ren S Wu S. Altered endocrine and autocrine metabolism of vitamin D in a mouse model of gastrointestinal inflammation. Endocrinology. (2008) 149:4799–808. 10.1210/en.2008-0060 18535110 PMC2582909

[90] Uno JK Kolek OI Hines ER Xu H Timmermann BN Kiela PR. The role of tumor necrosis factor α in down-regulation of osteoblast phex gene expression in experimental murine colitis. Gastroenterology. (2006) 131:497–509. 10.1053/j.gastro.2006.05.020 16890604

[91] Ensrud KE Barbour K Canales MT Danielson ME Boudreau RM Bauer DC. Renal function and nonvertebral fracture risk in multiethnic women: the Women's Health Initiative (WHI). Osteoporos Int. (2012) 23:887–99. 10.1007/s00198-011-1667-1 21625880 PMC3643305

[92] Yuan FL Li X Lu WG Xu RS Zhao YQ Li CW. Regulatory T cells as a potent target for controlling bone loss. Biochem Biophys Res Commun. (2010) 402:173–6. 10.1016/j.bbrc.2010.09.120 20920469

[93] Stagi S Cavalli L Iurato C Seminara S Brandi ML De Martino M. Bone metabolism in children and adolescents: main characteristics of the determinants of peak bone mass. Clin Cases Miner Bone Metab. (2013) 10:172–9. 24554926 PMC3917578

[94] Hanna MH Brophy PD. Metabolomics in pediatric nephrology: emerging concepts. Pediatr Nephrol. (2015) 30:881–7. 10.1007/s00467-014-2880-x 25027575 PMC4297580

